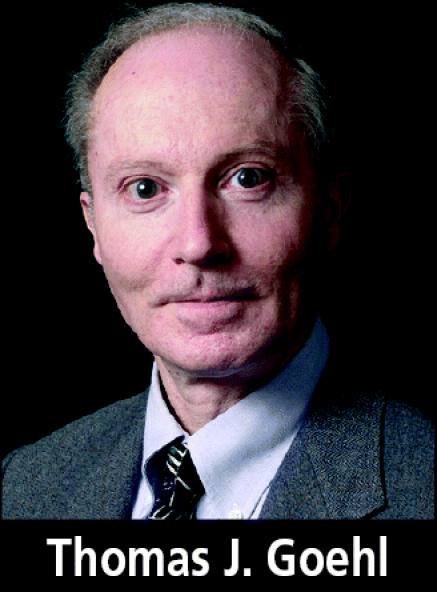# Editorial: Embracing Scrutiny

**DOI:** 10.1289/ehp.112-a788

**Published:** 2004-10

**Authors:** Thomas J. Goehl

**Affiliations:** Editor-in-Chief, *EHP*, Research Triangle Park, North Carolina, E-mail: goehl@niehs.nih.gov

Scientists are accustomed to scrutiny such as experimental protocol reviews, oversight on research conduct, and critiques provided during the peer-review publication process.

We especially anticipate the post-publication period during which others attempt to reproduce, validate, and then build on our work. All this scrutiny is basic to establishing the credibility of our findings. We therefore should not be put off by the greater attention now being focused on full disclosure of competing financial interests. What is essential to us as scientists is credibility. If our work is to contribute to the scientific and medical knowledge base, full disclosure is just one more process that must be embraced to establish and maintain the credibility of scientific and medical research.

“The potential for conflict of interest can exist whether or not an individual believes that the relationship affects his or her scientific judgment” (Davidoff et al. 2001); this quote should be the rule upon which authors lean when deciding on the necessity of providing a financial interest disclosure. Authors should also realize that disclosing financial support does not automatically diminish the credibility of the research. However, failure to disclose a competing financial interest that is subsequently discovered immediately opens the authors to questions about objectivity.

The need for full disclosure has become even more compelling as commercial organizations provide an increasing percentage of research support. Recognizing the growing importance of full disclosure, *EHP* clarified its policies in 2003. We feel that our disclosure requirements, which focus only on competing financial interests, are clear. However, a recent survey of the disclosure statements of some of our authors raised doubts about compliance.

The Center for Science in the Public Interest (CSPI 2004) surveyed four top medical and scientific journals: the *New England Journal of Medicine*, the *Journal of the American Medical Association* (*JAMA*), *EHP*, and *Toxicology and Applied Pharmacology*. The author of the report, Merrill Goozner, was quoted in *USA Today* (Davis 2004) as saying that “these journals were picked because they have the best policies.”

The CSPI investigative study covered December 2003 through February 2004, during which time *EHP* published 37 scientific studies. In a letter to *EHP*, Goozner (2004) stated that

Only 2 of the studies indicated they were funded by industry, and 2 studies included conflict of interest disclosure statements for at least some of the authors. … The CSPI investigated the first and last authors involved in the 35 studies who did not disclose conflicts of interest. Our investigation revealed at least 3 articles (8.6%) where either the first or last authors should have disclosed conflicts in accordance with the disclosure policy.

A fourth article was noted, but not included, because the issue identified would require a very strict interpretation of conflict of interest policies.

In an e-mail to Goozner, I expressed *EHP* ’s gratitude for the work done by the CSPI to help *EHP* achieve its goal of full disclosure of competing financial interests. I mentioned that we have a standing policy that encourages our readership to scrutinize disclosure issues. I further noted the difficulty that journal editorial offices would have if we undertook the task of checking on the financial interests of each of our authors. I promised Goozner that we would discuss this issue with our editorial board members and publish his letter along with responses that we would solicit from the named authors. These letters appear in this issue of *EHP* beginning on page A794*.*

The authors named by the CSPI (2004) have provided explanations for why they did not provide disclosure to *EHP*. After careful review of all the responses and discussions with our editorial board members, I am confident that there were no willful attempts to hide any competing financial interests. I judge that the authors named in the CSPI report (CSPI 2004) have made good faith efforts to comply with *EHP* disclosure policy.

However, lessons learned from examination of the four cases identified in the CSPI report (CSPI 2004) do provide guidance for future authors. In reporting affiliations, authors must ensure that they see the final formatted manuscript before submission. In deciding how in-depth their funding sources should be investigated, authors are expected to make a diligent effort to identify sources of funding and report that information. However, when funds come from a funding group that combines contributions from multiple sources, a failure to note a minor contributor is understandable. The probability is low that this minor percentage could impact the research findings or the personal finances of an author. Another clear requirement is that any relationship that could be perceived to have the potential for improperly influencing an author’s research should always be reported. In regard to patent disclosures, only existing, relevant patents issued before submission of a paper need to be disclosed. The issue of disclosure is quite complex. I counsel authors to always err on the side of caution. When in doubt, report!

Considering the issues raised by the CSPI report (CSPI 2004), we feel that it is appropriate for *EHP* to continue to update our disclosure policy. We now clearly instruct our authors to err on the sign of caution, and we have added the admonition that authors are to disclose all competing financial interests that might in any way be perceived as representing a competing financial interest. As has been our practice, *EHP* will continue to publish all disclosures made by our authors.

Our previous policy did not outline specific punitive measures that would be taken when our policies are violated. Because we feel that full disclosure is an absolute requirement, we are now adding clear consequences for any ethical violations. From now on, we will impose a 3-year ban on publication on authors who willfully fail to disclose a competing financial interest. Implementation of the ban will be made in consultation with our editorial board. If complete disclosure of possible conflicts would have caused the journal to have rejected the manuscript, the paper will be retracted. If the paper is not retracted but an ethical omission has occurred, an Expression of Concern will be written, published in the journal, and added to the online version of the article.

Once again, I encourage the scientific community to embrace scrutiny of our competing financial interests as we embrace the scrutiny of our research. Full disclosure is in the best interest of the individual scientists, the journals, and society, which must have complete faith that our research is not only of the highest quality but also is open, honest, and unbiased.

## Figures and Tables

**Figure:**